# Primary signet‐ring cell carcinoma of the lung: A report of seven cases

**DOI:** 10.1111/1759-7714.13614

**Published:** 2020-08-19

**Authors:** Yi Wang, Yan Wang, Jialong Li, Guowei Che

**Affiliations:** ^1^ Department of Thoracic Surgery, West China Hospital Sichuan University Chengdu China

**Keywords:** Case report, lung, signet‐ring cell carcinoma

## Abstract

Signet‐ring cell carcinoma (SRCC) is an extremely rare subtype of adenocarcinoma with aggressive behavior that usually occurs in the gastrointestinal tract, prostate or breast. In this study, we described the clinicopathological and prognostic characteristics of, and the therapeutic strategies for seven patients with primary SRCC of the lung from Sichuan University West China Hospital to enhance our understanding of this kind of tumor. According to the information presented herein, it is believed that patients with primary pulmonary SRCC can live for a long time if the cancer is diagnosed early and treated actively. However, more investigations are still needed due to the limited reports about primary SRCC of the lung.

**Key points:**

**Significant findings of the study:**

- Primary signet‐ring cell carcinoma of the lung is an extremely rare subtype of lung adenocarcinoma and is relatively highly invasive.

**What this study adds:**

- Early diagnosis and positive treatment lead to a good prognosis for patients with signet‐ring cell carcinoma.

## Graphical abstract

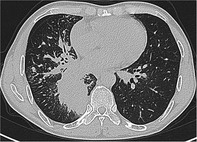

Primary signet‐ring cell carcinoma of the lung is an extremely rare subtype of lung adenocarcinoma and relatively highly invasive. Early diagnosis and positive treatment could bring a good prognosis to this kind of patients

## Introduction

Signet‐ring cell carcinoma (SRCC) is a special type of mucin‐secreting adenocarcinoma that contains abundant intracytoplasmic mucin and pushes the nucleus to one side of the cell.^1^ It originates from the undifferentiated stem cells of the mucosal lamina propria and is characterized by rapid growth, poor differentiation, diffuse infiltration and difficult early diagnosis. In addition, it often appears in the gastrointestinal tract, breast, bladder and prostate, and most patients with SRCC have a poor prognosis. SRCC in the lungs, which is extremely rare, mostly metastasizes from gastric cancer through the lymphatic pathway. In 1989, Kish *et al*. found that SRCC could appear as a primary tumor of the lung; the researchers reported five cases of primary pulmonary SRCC with abundant extracellular mucin secretion.^1^ Primary SRCC of the lung was then identified in the third edition of the World Health Organization classification system as a histological subtype of lung cancer.^2^ At present, the relevant literature about primary SRCC of the lung mostly comprises case reports or small‐scale retrospective clinical analyses,^3^, ^4^ with the exception of two relatively large studies.^5^, ^6^ One study was conducted by Ou *et al*. in 2010, wherein they analyzed 262 pulmonary SRCC patients.^5^ The other study was conducted by Wu *et al*. wherein they reviewed 24 171 SRCC cases from the Surveillance, Epidemiology and End Results (SEER) database and reported the clinicopathological and prognostic parameters of 738 (3.1%) of those cases.^6^ Due to the limited number of reports about primary SRCC of the lung, there are still challenges to standardizing the clinical diagnosis and treatment of this kind of carcinoma.

Here, we described the clinicopathological and prognostic characteristics of seven primary pulmonary SRCC patients from our center to share our experience with the treatment of this type of tumor.

## Case report

**Figure 1:**
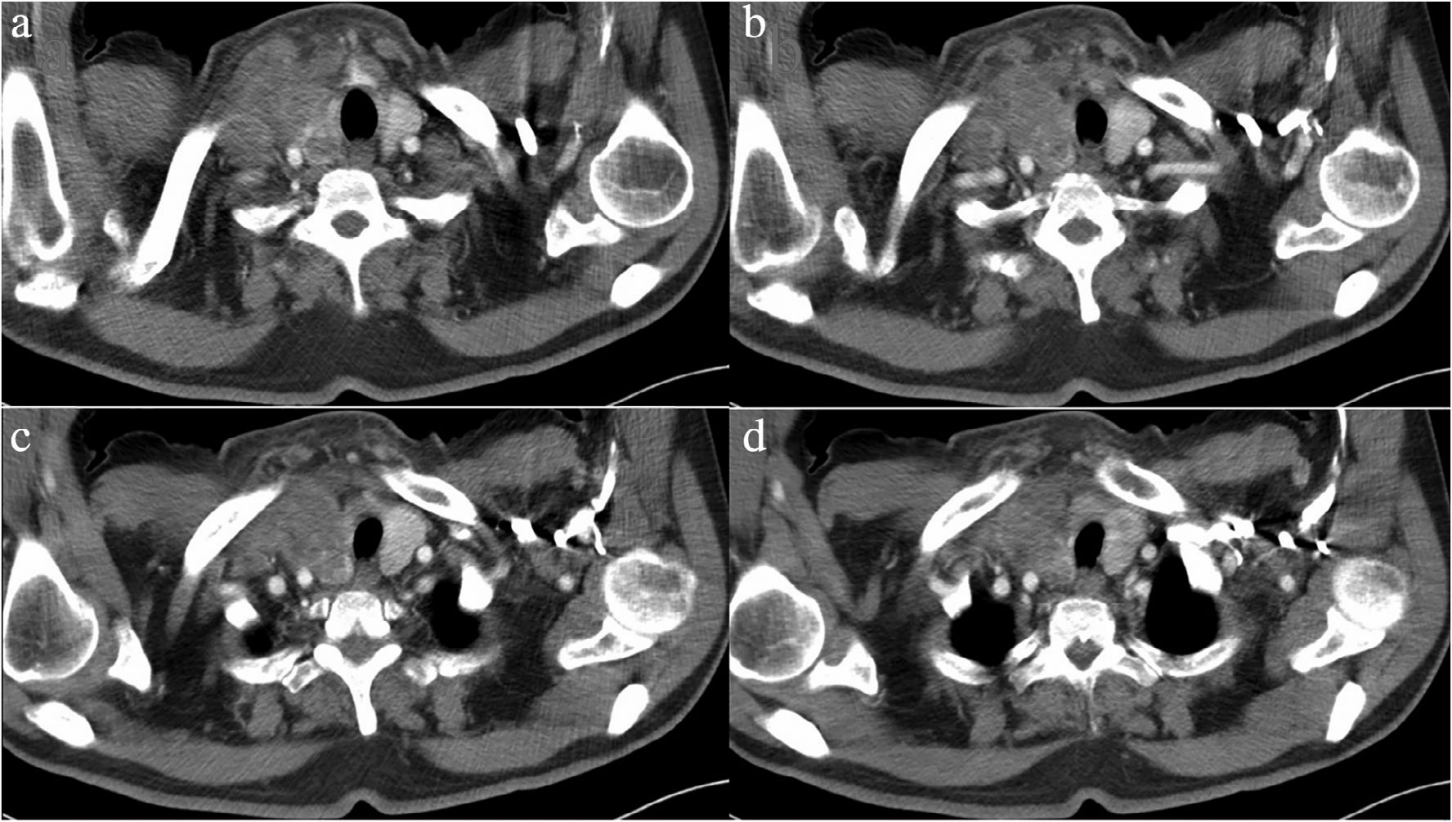
Cervical computed tomography (CT) scan revealed a 4.5 × 3.7 cm mass in the right thyroid gland of patient No. 3.

**Figure 2:**
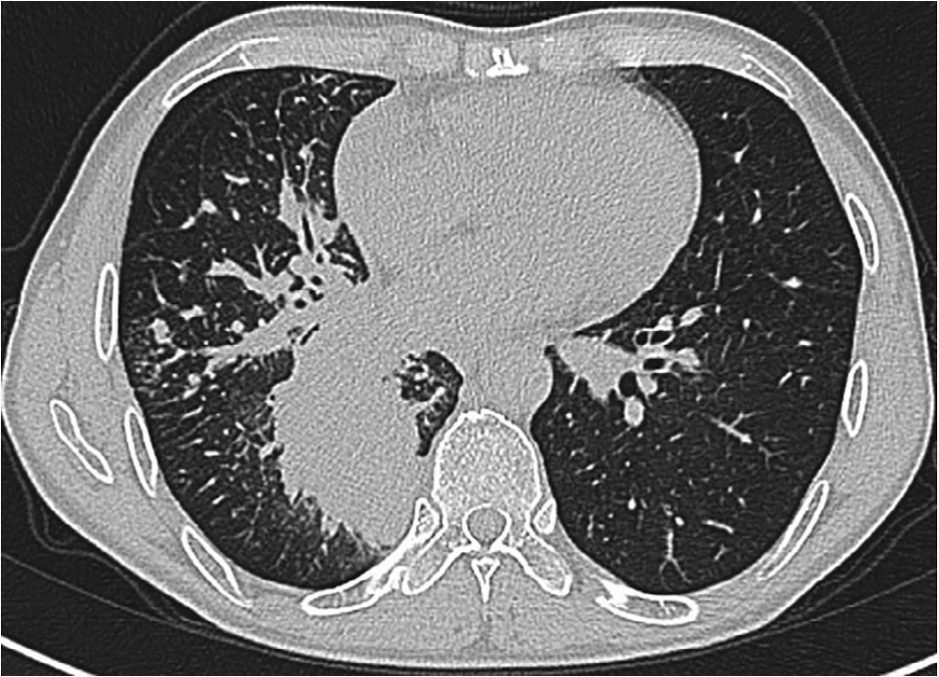
Chest computed tomography (CT) scan revealed an 5.8 × 4.4 cm mass in the right inferior lobe of patient No. 1.

**Figure 3:**
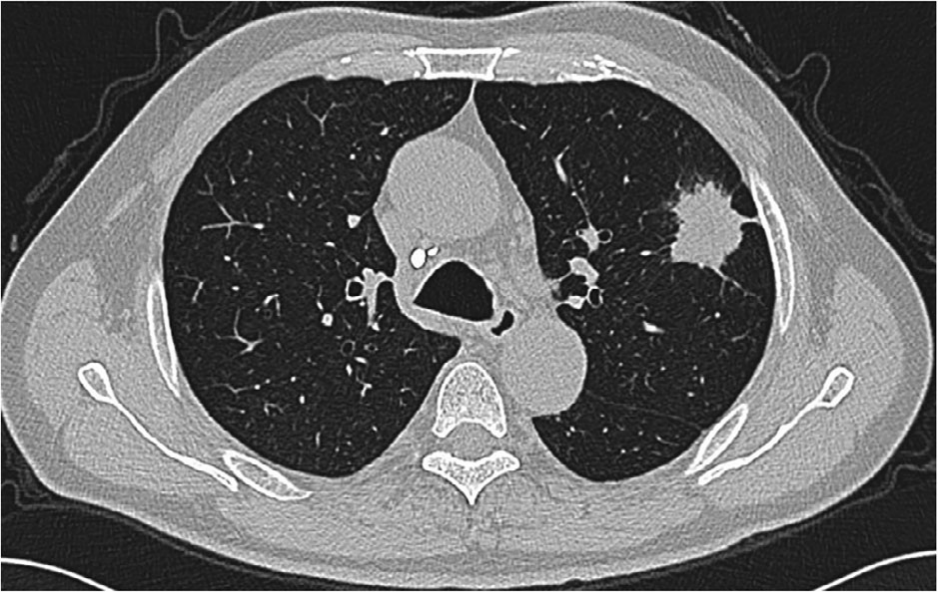
Chest computed tomography (CT) scan revealed a 3.8 × 3.4 cm mass in the left upper lobe of patient No. 4.

**Figure 4:**
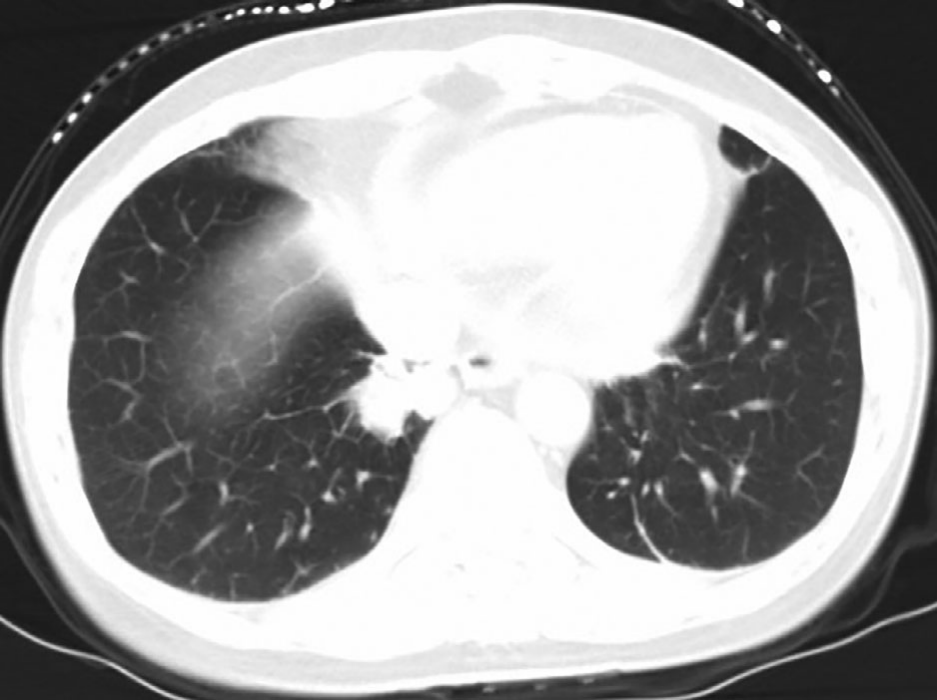
Chest computed tomography (CT) scan revealed a 2.6 × 2.3 cm nodule in the right inferior lobe of patient No. 5.

The data from seven patients with primary lung adenocarcinoma with signet‐ring cell components diagnosed from 2010 to 2018 were reviewed, including four male patients and three female patients. The median age of the patients was 62 (ranging from 47 to 74), with a median follow‐up time of 27 months (ranging from 2 to 86 months). Five patients were still alive at the end of the follow‐up (20 June 2020), and the other two patients died of this disease. Other patients except patient No. 6 had symptoms such as coughing. All male patients had a smoking history, with a smoking index ranging from 100 to 900, but all female patients were nonsmokers. Regarding the location of the masses, notably, patient No. 3 was found to have a large mass in the right thyroid, and no obvious lesions in the lungs were observed, except for some nodules (Fig 1). Patient Nos. 1, 4 and 5 had masses in the lungs according to computerized tomography (CT) performed in our hospital (Figs 2, 3 and 4). Two of the seven patients were diagnosed with eighth edition tumor‐node‐metastasis (TNM) stage IB, and four patients had distant metastases at the time of diagnosis. (Table 1).

**Table 1:** Clinicopathological parameters of seven primary carcinomas with SRCC components

No.	Age	Gender	Follow‐up (month)	Symptom	Comorbidity	Smoking index	Location	T	N	M (location)	Eighth edition TNM stage	Differentiation	SRCC (%)	Treatment	TTF‐1	NapsinA	CK7	CK20	ALK	ROSI	EGFR
1	47	M	Alive (24)	Cough	None	360	RLL	4	3	1b (brain)	cIVA	**‐**	<50%	TT+CT (bevacizumab + paclitaxel + carboplatin for six cycles, bevacizumab for two cycles)	+	+	+	‐	‐	+	‐
2	50	M	Alive (27)	Cough and pain in left chest and back	None	900	LUL	4	2	0	pIIIB	Low‐moderate	>50%	Surg					+	‐	
3	71	M	Dead (2)	Headache	Diabetes and Hypertension	800	BMNs	‐	2	1b (thyroid)	pIVA	Low	<50%	None	+		+	‐			
4	74	M	Alive (86)	Cough and expectoration	None	100	LUL	2a	0	0	pIB	Low‐moderate	>50%	Surg+CT (pemetrexed for six cycles)					‐		‐
5	58	F	Alive (55)	CoughCough	None	0	RLL	2a	3	1c (liver and brain)	cIVB	Low	<50%	CT+TT (etoposide + cisplatin for two cycles, paclitaxel + carboplatin for two cycles, pemetrexed + bevacizumab for two cycles, apatinib, crizotinib)	+	+	+	‐	‐	‐	‐
6	62	F	Alive (55)	None	Diabetes	0	RUL	2a	0	0	pIB	Low‐moderate	<50%	Surg+CT (pemetrexed + cisplatin for one cycle)	+	+	+				
7	63	F	Dead (19)	Air tightness after exercise, cough and hoarseness	Hypertension	0	LLL	‐	2	1c (liver, spleen and bone)	pIVB	Low	‐	CT+TT (docetaxel + oxaliplatin for five cycles, gefitinib, pemetrexed + cisplatin for six cycles)							

CT, chemotherapy; BMN, bilateral multiple nodules; EGFR, epidermal growth factor receptor; F, female; LUL, left upper lobe; LLL, left lower lobe; M, male; RLL, right lower lobe; RUL, right upper lobe; SRCC, signet‐ring cell carcinoma; Surg, surgery; TNM, tumor‐node‐metastasis; TT, targeted therapy; TTF‐1, thyroid transcription factor 1.

All patients showed low or low‐moderate differentiation status except for patient No. 1, whose differentiation information was not available. Signet‐ring cell components were predominant in four patients. The results of immunohistochemical indicators are shown in Table 1. Patients with primary SRR of the lung tend to show positive TTF‐1, NapsinA and CK7 expression and negative CK20 and EGFR expression (Table 1).

Three of the seven patients without distant metastasis (Nos. 2, 4 and 6) underwent surgery, and two patients with TNM stage IB (Nos. 4 and 6) received adjuvant chemotherapy. Patient No. 2 underwent left upper lobectomy, left pulmonary artery sleeve formation and systemic lymph node dissection and was diagnosed with pTNM IIIB after surgery. Three patients (Nos. 1, 5 and 7) with distant metastasis received chemotherapies and targeted therapies, and the detailed information is shown in Table 1. Unfortunately, patient No. 3 died of respiratory failure before receiving antitumor treatment.

## Discussion

To date, the literature about primary pulmonary SRCC mostly comprises case reports or small‐scale retrospective clinical analysis. Some clinicopathological and prognostic characteristics were not known until the publication of the research conducted by Ou *et al*. in 2010.^5^ They investigated the clinicopathological and prognostic features of 262 patients with primary SRCC of the lung compared to 50 089 lung adenocarcinoma patients. According to their results, primary pulmonary SRCC patients were younger, had a higher proportion of distant metastasis and poor differentiation, and had significantly shorter overall survival (OS) than adenocarcinoma patients. The signet‐ring cell component was an independent risk factor for the prognosis of lung adenocarcinoma patients (hazard ratio [HR] = 1.214, 95% confidence interval [CI]: 1.068–1.381, *P* = 0.003). However, the researchers did not conduct subgroup analysis to explore the value of particular treatment strategies for primary pulmonary SRCC patients, especially for TNM stage IV patients who were inoperable.

In 2018, another study of primary pulmonary SRCC by Wu *et al*. analyzed a total of 738 cases.^6^ They found that most primary pulmonary SRCC patients had a poor differentiation status, had distant metastasis at the initial diagnosis and received less aggressive treatment. In addition, the five‐year cancer‐specific survival (CSS) rate was only 11% with a median CSS of 6 months, which indicated that the prognosis for most patients with primary pulmonary SRCC was poor.

Compared with previously reported results, some novel information could be obtained from our study. For primary pulmonary SRCC patients with distant metastasis, active treatment is also necessary. Patient No. 5 received chemotherapies and targeted therapies and survived for 55 months after being diagnosed; however, he was not particularly sensitive to chemotherapy, so his therapy strategy was changed twice. For patients without distant metastasis, surgical therapy may be valuable because patients No. 2, 4 and 6 all had relatively good prognoses.

In conclusion, in this study, we described the clinicopathological and prognostic features of seven patients with primary SRCC of the lung and attempted to reveal valuable information about the treatment strategy. However, more investigations are still needed to fully understand this kind of tumor.

## Disclosure

The authors have no conflicts of interest to declare.
